# Quality of life of children during distraction osteogenesis: a comparison between intramedullary magnetic lengthening nails and external fixators

**DOI:** 10.1007/s00264-022-05399-w

**Published:** 2022-04-07

**Authors:** Mohamed Hafez, Nicolas Nicolaou, Amaka Offiah, Bright Offorha, Stephen Giles, Sanjeev Madan, James A. Fernandes

**Affiliations:** 1 Paediatric Limb Reconstruction Unit, Sheffield Children’s Hospital, Western Bank, Sheffield, S10 2TH UK; 2 Oncology and Metabolism Department, Medical School, Sheffield University, Sheffield, UK; 3 Sheffield University, Sheffield, UK; 4 School of Health and Related Research, Sheffield University, Sheffield, UK

**Keywords:** Magnetic nails, Limb lengthening, External fixators, Quality of life

## Abstract

**Background:**

Distraction osteogenesis is a very demanding process. For decades, external fixation was the only reliable option for gradual deformity correction. Recently, intramedullary magnetic nails have gained popularity. This research aimed to assess the quality of life in children during gradual deformity correction using intramedullary lengthening nails compared to external fixation.

**Method:**

Prospective analysis included children who had gradual lower limb deformity correction between 2017 and 2019. Group A included children who had magnetic lengthening nails; patients in group B had external fixation devices. Child health utility 9D (CHU- 9D) and EuroQol 5D youth (EQ- 5D-Y) were used to measure the quality of life at fixed points during the distraction osteogenesis process. The results were used to calculate the utility at each milestone and the overall quality of life adjusted years (QALYs).

**Results:**

Thirty-four children were recruited, group A had 16 patients, whilst group B had 18 patients. The average ages were 16.0 years and 14.7 years for groups A and B, respectively. Group A patients reported significantly better utility compared to group B. This was observed during all stages of treatment (*P* = 0.00016). QALYs were better for group A (0.44) compared to group B (0.34) (*P* < 0.0001).

**Conclusion:**

The quality of life was generally better in group A compared to group B. In most patients, the health utility progressively improved throughout treatment. In the same way, QALYs were better with the lengthening nails compared to external fixators. The magnetic lengthening devices (PRECICE nails) which were used in this research were recently relabelled to restrict their applications in children; this study was conducted before these restrictions.

## Background

Significant lower limb deformity and limb length discrepancy LLD (> 2 cm) are estimated to affect 1:2000 children [1]. If left untreated, they can lead to gait abnormalities and pain. The abnormalities in appearance and function may make it difficult for affected children to participate in sports, educational and leisure activities. As a result, psychological and emotional difficulties are reported to be more common in children with lower limb deformities [2].

Traditionally, external fixators were essential for distraction osteogenesis (DO) [3]. External fixators require daily care and modification of lifestyle to reduce the risks of pin site infection and adjacent joint stiffness. Patients need special training to be able to make the daily adjustments of the rods/struts required to produce the planned correction. External fixation devices can be cumbersome and are commonly associated with complications [4]. The nature of external fixation, prolonged hospitalisation, multiple operative procedures and increased rates of complications can result in significant psychological and emotional problems in children [4]. In one series, half the children who had Ilizarov fixators had moderate to a severe worsening of their mental health and suicidal thoughts [5]. These psychological and emotional abnormalities were thought to be reversible when the children were reassessed following the removal of the devices [2, 5–7].

The frequent complications of external fixators in addition to the emphasis on quality of life and the emotional well-being of patients led to the development of fully implantable motorised lengthening nails [3]. PRECICE lengthening nails (NuVasive Specialized Orthopedics Inc. Aliso Viejo, CA, USA) are magnetic telescopic titanium intramedullary lengthening nails. The nails are activated by external remote control (ERC) to produce the required distraction. Lengthening nails were reported to have better clinical outcomes than external fixators for limb lengthening in children [8, 9]. Lengthening nails are more expensive than external fixators. This added cost of lengthening nails was argued to be in exchange for a better quality of life during the lengthening process [10, 11]. Although this may be true, no studies have compared the quality of life between lengthening nails and external fixators during treatment.

Health-related quality of life (HR-QOL) measures have a vital role in contemporary healthcare. Validated HR-QOL tools are used for utility analysis. The utility can be used to calculate the quality-of-life adjusted years (QALYs), which is extremely useful for the allocation of health care resources. This study compares the reported quality of life in children whilst they were undergoing treatment with either lengthening nails or external fixation.

## Methods

Approval of this research was granted by the local research and development department. This was a prospective study. Inclusion criteria consisted of the following: (1) age between nine and 17 years; (2) lower limb reconstruction (lengthening and deformity correction, both acquired and congenital) with lengthening nails or external fixators between 2017 and 2019. Informed consent/assent was obtained from eligible children and their carers. Patients who had external fixators for soft tissue correction only without bony procedures and those who refused to participate in the research were excluded. Patients were divided into two groups: group A included children who had PRECICE lengthening nails (NuVasive Specialized Orthopedics Inc. Aliso Viejo, CA, USA), whilst group B included children who had external fixators whether monolateral or circular types.

The patients were asked to fill the HR-QOL questionnaires during their outpatient appointments on three occasions: the first was during the distraction stage (1 month post-operative), the second was during the early consolidation stage (3 months post-operative) and the third during the late consolidation/healed stage (9 months post-operative or before frame removal if removed earlier than 9 months). CHU-9D instrument was used as the primary outcome measure, whilst the EQ-5D-Y instrument was used as a secondary outcome. The institutions which developed these outcomes measures approved their use in this research. The utility was generated from each completed questionnaire. QALY was then calculated using the utilities at the different time intervals.

In our protocol, early weight-bearing was started following external fixation procedures, whilst non-weight bearing was advised following lengthening nail procedures until adequate bone formation. Distraction was commenced at days five to seven post-operatively at a rate of 1 mm/day (0.25 mm four times/day for external fixation, and 0.33 mm three times/day with lengthening nails). The distraction rate was adjusted according to the quality of bone formation, tolerance of the patients and nearby joints’ range of motion. In our unit, magnetic lengthening nails were not used for tibial lengthening. Therefore, the tibial lengthening patients were recruited to the external fixators group. Meanwhile, the femoral lengthening patients had the opportunity (when possible) to choose between lengthening nails and external fixators, and they were treated accordingly.

### Statistical analysis

A pilot study included 8 patients in each group. The CHU-9D utility was used for the power calculation to determine the sample size. To calculate a mean difference of 0.13 (SD) between groups, a sample size of 16 patients with completed scores per group were required (80% power, 5% significance, 2-tailed analysis). To accommodate for 20% missing CHU-9D values (non-differential between the treatment groups), 20 patients were recruited. Patients with incomplete primary outcomes (CHU-9D) were excluded from the study, whilst the patients with missing EQ-5D-Y were included to analyse their CHU-9D results. Multiple statistical tests were used. Mean, standard deviation (SD), median and interquartile range (IQR) were used for descriptive analyses of continuous outcomes, whilst frequency and percentage were reported for categorical outcomes. Linear mixed model regression was used for the analysis of utility. Wilcoxon rank-sum test and Friedman non-parametric tests were used for the analysis of the dimensions of the HR-QOL instruments. A linear regression model was utilised for the analysis of QALYs. For all statistical analyses, *P* value < 0.05 was regarded as significant.

## Results

Each group included 20 patients. After excluding the patients with missing CHU-9D responses, group A included 16 children, whilst group B included 18 children. All the included children in the final sample completed the CHU-9D instruments at the three identified time points. Eight children in group A had missing responses to one or more domains of the EQ-5D-Y instrument on one or more occasions. Meanwhile, all the patients in group B filled the EQ-5D-Y questionnaires fully. The mean age was 16.06 (SD 1.8) years and 14.67 (SD 2.5) years for groups A&B respectively. Group A included ten males whilst group 12 male patients. The femur was the involved segment for all the patients in group A, whilst in group B there were 14 tibial and four femoral segments.

Group A patients reported significantly better scores in all domains of CHU-9D (Table 1). Apart from the domains ‘sad and annoyed’, all the domains showed significant improvement over time in group A. In group B, all the domains showed progressive improvement over time except for the ‘annoyed’ domain.

**Table 1:** Median Chu9D comparison (responses) and the *P* value of each dimension

	Group A (Lengthening nail)			Group B (External fixator)			*P* value
	Distraction	Early consolidation	Late consolidation	Distraction	Early consolidation	Late consolidation	
Study sample size	16			18			
Worry	1	1	1	2	1	1	0.0046
Sad	1	1	1	2	2	1	0.0001
Pain	3	1	1	3	2	2	< 0.0001
Tired	2	2	1	3	2	2	0.00023
Annoyed	1	1	1	2	2	1	< 0.0001
School	2	1	2	3	2	2	0.0041
Sleep	2	1	1	3	2	1	0.016
Daily routine	2	1	1	4	3	2	< 0.0001
activities	5	4	2	5	4	4	0.0039

The mean overall utility was 0.85 (SD = 0.13) for group A and 0.70 (SD = 0.17) for group B. For the unadjusted model (model with only treatment group as a covariate), the mean difference in utility between the two groups was significant (95% CI: 0.08 to 0.22, *P* value = 0.0002) and remained significant after all variables such as age and gender had been adjusted for (*P* = 0.003), in favour of group A. Age and gender differences were not associated with any significant difference in utility (*P* = 0.2 and *P* = 0.08 for age and gender respectively).

**Fig. 1:**
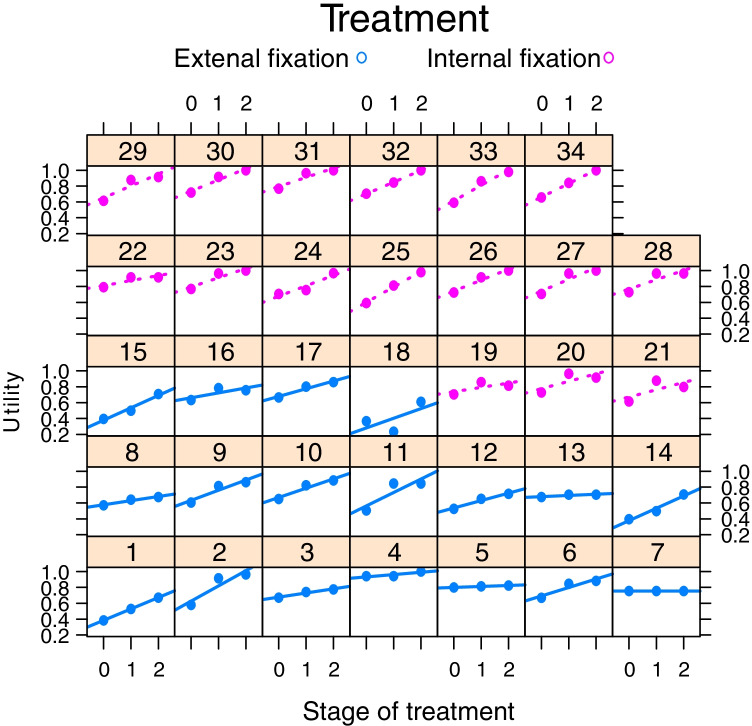
Comparison of time trend plots for each patient utility over time between the two treatment groups (stage 0 is a distraction, 1 and 2 are for early and late consolidations)

**Fig. 2:**
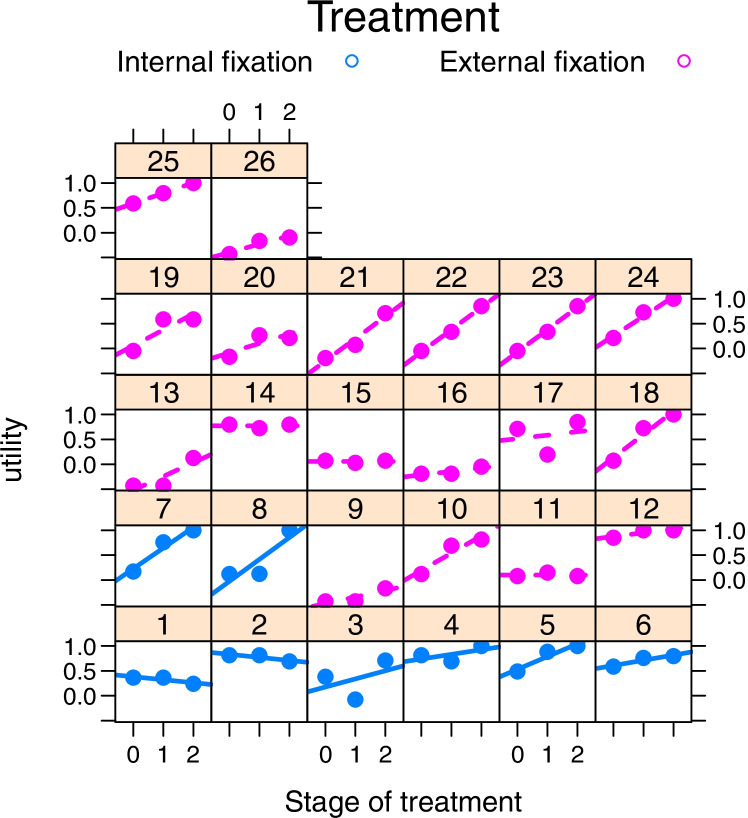
Time trend plot for EQ-5D-Y utility for each patient in the two treatment groups. (Stage 0 is at distraction, 1 and 2 are for early and late consolidations)

**Fig. 3:**
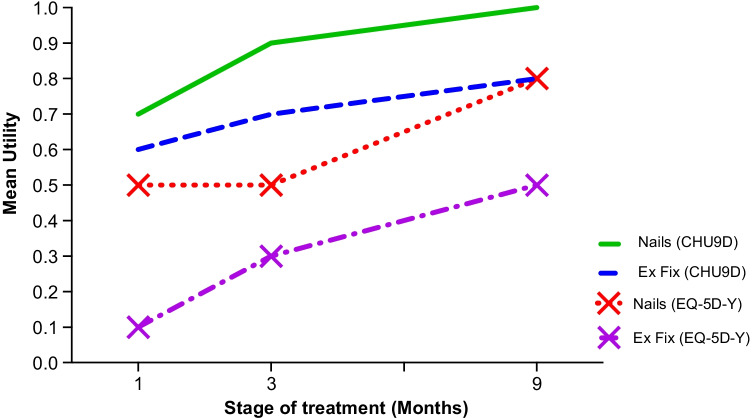
Line graphs of EQ-5D-Y, CHU9D mean utilities for both nails and external fixators

Within the same group, the utility showed a progressive increase over time. Table 2 outlines the means of the CHU-9D and EQ-5D-Y utilities at each time point. Figure 1 summarises the CHU-9D utilities for all the patients at the different time points. Figure 2 summarises the EQ-5D-Y utilities for all the patients at the different time points. Figure 3 compares the mean CHU-9D and EQ-5D-Y utilities of the two groups.

**Table 2:** Summary of CHU-9D and EQ-5D-Y utility at different time points for the two treatment groups

CHU-9D utility						
	Group A (Lengthening nails)			Group B (External fixation)		
Stage of treatment	*n*	Mean	SD	*n*	Mean	SD
Distraction	16	0.69	0.06	18	0.60	0.15
Early	16	0.89	0.06	18	0.71	0.18
Late	16	0.95	0.07	18	0.79	0.11
EQ-5D-Y utility						
	Group A (Lengthening nails)			Group B (External fixation)		
Stage of treatment	*n*	Mean	SD	*n*	Mean	SD
Distraction	8	0.47	0.31	18	0.08	0.46
Early	8	0.54	0.32	18	0.30	0.45
Late	8	0.80	0.33	18	0.54	0.44

Using the CHU9D instrument, the mean QALYs were 0.44 (SD = 0.02) for group A and 0.34 (SD = 0.07) for group B. The difference in QALYs was significant (95% CI: 0.04 to 0.12, *P* = 0.00015) and remained significant (*P* = 0.00016) when age and gender were adjusted for, and in favour of group A. QALYs were not significantly different between different ages and genders (*P* = 0.49 and 0.14 for age and gender respectively). In the same way, the QALY which was generated from EQ-5D-Y data was higher in group A (0.30) compared to group B (0.17).

## Discussion

External fixation is an established method of deformity correction. Limb reconstruction centres developed extensive rehabilitation programmes to reduce complications and improve patients’ experience [12]. However, multiple researchers reported the negative psychological and emotional effects of these devices during the limb lengthening process [5]. Lengthening nails were introduced to improve the patient QOL during that phase [3]. The psychological problems were reported to be reversible following the removal of the external fixator [6]; therefore, we focused this research on the comparison of QOL during the lengthening session.

Until recently, there were no reliable or validated instruments for measuring patient-reported health status in children, particularly preference-based measures (PBMs) [13] that allow for the calculation of QALYs. CHU-9D and EQ-5DY are both PBMs. EQ-5DY (EuroQol, Rotterdam, Netherlands) was developed from the existing adult instrument (EQ-5D). EQ-5D-Y has the same five dimensions as the EQ-5D but with language adaptation for children. EQ-5D-Y has five questions for five domains. Each question has three answers, with no limitation to QOL as number 1 and significant limitation as option 3. It was argued that further work on EQ-5D-Y is required to confirm the content validity in children [14]. CHU-9D (Sheffield University, Sheffield, UK) was primarily developed to be used for children rather than being a modified version of an adult instrument [14]. CHU-9D contains nine dimensions (worried, sad, pain, tired, annoyed, schoolwork/homework, sleep, daily routine and activities). Each domain is represented by a question that has five answers, numbered 1–5, with number 1 being normal and number 5 being the worst for that dimension QOL. The advantages of CHU-9D include a short recall period (today), suitable for use in children seven to 18 years, easy and quick to be filled with two minute completion time, and it can be completed by patient or proxy [13].

Multiple studies compare the patients reported outcomes between lengthening nails and external fixators [10, 11, 15]. It was concluded that lengthening nails offered better patient satisfaction compared to external fixators. However, none of these studies used a validated outcome tool. As a result, this study used child-specific, preference-based validated HR-QOL instruments. Recall bias was identified in previous research. The patients were asked to comment on their experience of the fixation devices after the removal of the devices. The prospective design of this study, assessment of QOL at different timelines and the short recall time of CHU-9D all help to avoid recall bias.

The patients who were treated with lengthening nails reported less pain compared to external fixation. Common adverse events with external fixators such as pin site infection and pin loosening might explain the increased pain with external fixators. Pain could be the main reason for the increased anxiety, sadness, tiredness, sleep problems, annoyance and lack of independence with external fixators. The large sizes of external fixators and the inability to conceal them might cause low self-esteem which may be another factor for the inferior quality of life with these devices.

The improvement of quality of life throughout the treatment was expected. QOL was the lowest throughout treatment during the distraction phase. The relatively low QOL during the distraction phase compared to later stages of treatment can be explained by the ongoing distraction causing pain, apprehension and anxiety. Patients require some time to adjust to the new device and their new body image, especially with external fixators. Moreover, patients normally require more frequent visits to the outpatient department during the distraction phase which might cause anxiety. Unsurprisingly, patients reported better QOL during the late consolidation phase. The lengthening nails group reported the utility to be almost of perfect health (0.95). This might be related to the advanced bone healing in this stage which enabled patients to participate in more activities.

The missing data for eight patients for the EQ-5D-Y in the lengthening nail group limited the potential of this study to report on the correlation between the two instruments; however, this was not the main aim of the research. Another limitation is that the participants were not matched. In this study, the external fixator group participants were younger than the lengthening nail patients. This may produce different responses to the questionnaires according to the priorities at certain ages. In the same way, the participants were not matched for gender; more males were included in the external fixation group. However, the reported regressing analysis suggested that age and gender do not have a significant effect on the utility and QALYs. The diagnosis and the complexity of deformities were not matched between the two groups; this might have led to a difference in the representation of complex patients in the two groups.

Recently, the magnetic lengthening nails were recalled by the manufacturer due to raised biocompatibility concerns [16]. The Medicines and Healthcare products Regulatory Agency (MHRA) followed that with a recommendation not to implant the magnetic lengthening devices in the UK. The biological risks were linked to the stainless-steel design of the new generation implant (STRYDE nails). It is hypothesised, the corrosion at the telescopic junction of the nail might have been responsible for the periosteal reaction, metallosis and pain which were reported with the STRYDE nails [17, 18]. These effects were not reported with the titanium PRECICE nails which were used in this study. In November 2021, Magnetic nails were relabelled to be not suitable for patients younger than 18 years [19]; this study focused on the period between 2017 and 2019.

## Conclusion

Lengthening nails offered better health utilities and QALYs for children during the distraction, early and late consolidation phases of distraction osteogenesis compared to external fixators. This is the only study assessing the quality of life of children during the distraction osteogenesis process using validated HR-QOL instruments.

This research presented the QALYs during limb lengthening and provides a basis for further economic evaluations of the different techniques of distraction osteogenesis in children.
